# Crystal structure of an unknown solvate of (piperazine-κ*N*){5,10,15,20-tetra­kis­[4-(benzo­yloxy)phen­yl]porphyrinato-κ^4^ *N*}zinc

**DOI:** 10.1107/S2056989016009269

**Published:** 2016-06-14

**Authors:** Soumaya Nasri, Khaireddine Ezzayani, Ilona Turowska-Tyrk, Thierry Roisnel, Habib Nasri

**Affiliations:** a Laboratoire de Physico-chimie des Matériaux, Faculté des Sciences de Monastir, Avenue de l’environnement, 5019 Monastir, University of Monastir, Tunisia; b Faculty of Chemistry, Wroław University of Technology, Wybrzeże Wyspiańskiego 27, 50-370 Wroław, Poland; c Centre de Diffractométrie X, Institut des Sciences Chimiques de Rennes, UMR 6226, CNRS–Université de, Rennes, 1, Campus de Beaulieu, 35042 Rennes Cedex, France

**Keywords:** crystal structure, zinc porphyrin, piperazine, hydrogen bonds, UV–visible spectra

## Abstract

The title compound, [Zn(C_72_H_44_N_4_O_8_)(C_4_H_10_N_2_)] or [Zn(TPBP)(pipz] (where TPBP and pipz are 5,10,15,20-tetra­kis­[4-(benzo­yloxy)phen­yl]porphyrinato and piperazine ligands respectively), features a distorted square-pyramidal coordin­ation geometry about the central Zn^II^ atom. This central atom is chelated by the four N atoms of the porphyrinate anion and further coordinated by a nitro­gen atom of the piperazine axial ligand, which adopts a chair confirmation. The average Zn—N(pyrrole) bond length is 2.078 (7) Å and the Zn— N(pipz) bond length is 2.1274 (19) Å. The zinc cation is displaced by 0.4365 (4) Å from the N_4_C_20_ mean plane of the porphyrinate anion toward the piperazine axial ligand. This porphyrinate macrocycle exhibits major *saddle* and moderate *ruffling* deformations. In the crystal, the supra­molecular structure is made by parallel pairs of layers along (100), with an inter­layer distance of 4.100 Å while the distance between two pairs of layers is 4.047 Å. A region of electron density was treated with the SQUEEZE [Spek (2015 ▸). Acta Cryst. C71, 9–18] procedure in *PLATON* following unsuccessful attempts to model it as being part of disordered *n*-hexane solvent and water mol­ecules. The given chemical formula and other crystal data do not take into account these solvent mol­ecules.

## Abstract 2

The mol­ecular structure of the piperazine[5,10,15,20-(tetra­phenyl­benzoate)porphyrinato-κ^4^ *N*]zinc(II) complex is composed of parallel pairs of layers with an inter­layer distance of 4.100 Å while the distance between two pairs of layers is 4.047 Å.

## Chemical context   

The Zn^II^ ion is one of the most prevalent metal ions as the metal center of a metalloporphyrin. Indeed, zinc porphyrin complexes provide simpler systems than those of iron, cobalt, or other *d* transition metals to evaluate the influence of a wide range of different ligands on the spectroscopic and structural properties of complexed porphyrins. The metal ion is unambiguously in the +II oxidation state; in most cases, four-coord­inate (porphyrinato) zinc complexes will accept one axial ligand to form complexes with a coordination number of five for the metal (Denden *et al.*, 2015 ▸). Nevertheless, zinc porphyrins with a coordination number of six for the metal have also been reported (Shukla *et al.*, 2000 ▸; Oberda *et al.*, 2013 ▸).
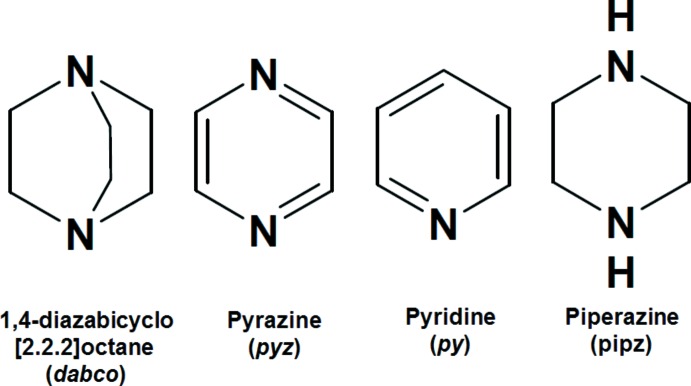
 
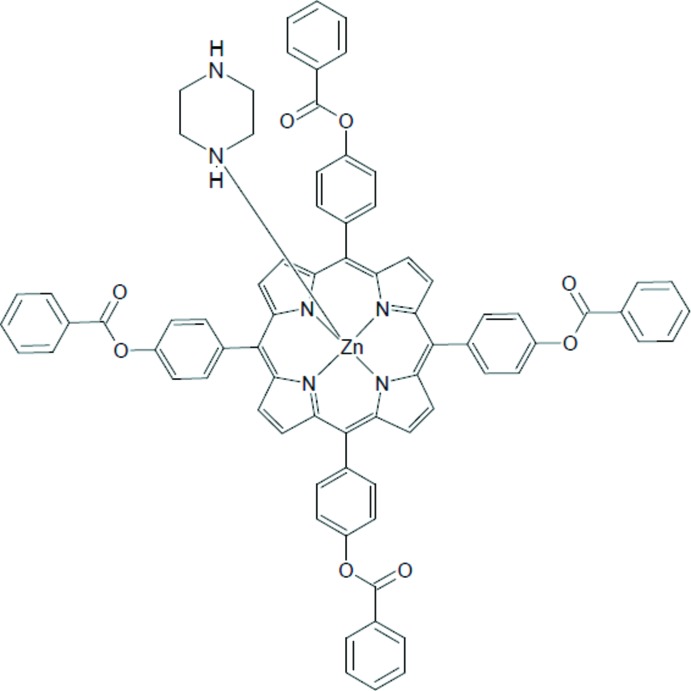


In the literature, an important number of zinc–pyridine (and substituted pyridines) metalloporphyrins have been reported, *e.g.* [Zn(TPP)(py)] (TPP = 5,10,15,20-tetra­phenyl­porphyrinato) (Devillers *et al.*, 2013 ▸). This is also the case for other related cyclic N-donor ligands such as dabco (1,4-di­aza­bicyclo­[2.2.2]octa­ne) and pyz (pyrazine), *e.g.* [Zn(OEP)(dabco)] (OEP = octa­ethyl­porphyrinato) (Konarev *et al.*, 2009 ▸) and [Zn(TPP)(pyz)] (Byrn *et al.*, 1993 ▸). Notably, to date no zinc–piperazine porphyrin structure has been reported in the literature. In this work, we have focused on the crystal structure and the UV–visible characterizations of the new zinc porphyrin title complex, namely the (piperazine){5,10,15,20-tetra­kis­[4-(benzo­yloxy)phen­yl]porphyrinato}zinc complex (I).

## Structural commentary   

**Figure 1:**
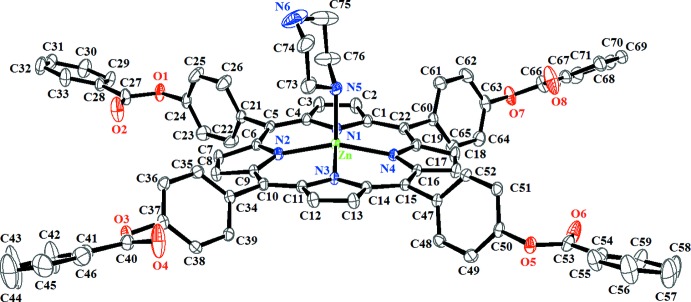
An *ORTEP* view of the mol­ecular structure of the [Zn(TPBP)(pipz)] complex with the atom-numbering scheme. Displacement ellipsoids are drawn at the 40% probability level. H atoms have been omitted for clarity.

**Figure 2:**
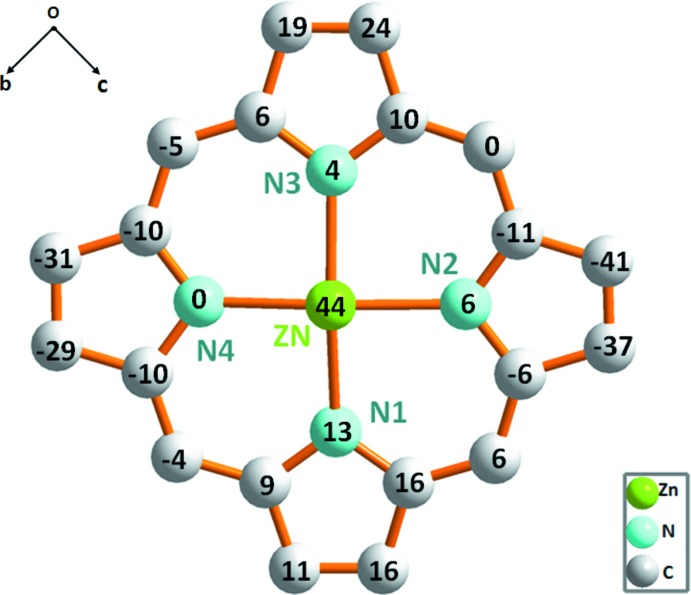
Formal diagram of the porphyrinate core illustrating the displacements of each atom from the 24-atom core plane in units of 0.01 Å.

The Zn^II^ cation is chelated by four pyrrole-N atoms of the porphyrinate anion and coordinated by a nitro­gen atom of the piperazine axial ligand in a distorted square-pyramidal geometry. The piperazine ligand adopts the usual *chair* conformation (Fig. 1 ▸). The Zn^__^N(pipz) bond length [2.1274 (19) Å] is considerably longer than the related non-porphyrinic zinc–pipz distances which are in the range 2.039 (3)–2.064 (2) Å (Suen *et al.*, 2002 ▸; Nguyen *et al.*, 2006 ▸) but shorter than that of the zinc–di­methyl­piperazine [{Zn(TPP})_2_(μ_2_-N,N′-di­methyl­piperazine)] [2.250 (2) Å; Konarev *et al.*, 2007 ▸]. The average equatorial zinc–N(pyrrole) distance (Zn—Np) is 2.078 (7) Å, which is close to those of related zinc metalloporphyrins of type [Zn(Porph)(*L*)] (Porph and *L* are a porphyrinato and a monodentate neutral ligand, respectively; Byrn *et al.*, 1993; Lipstman *et al.*, 2006 ▸). Fig. 2 ▸ is a formal diagram of the porphyrinato core atoms of (I) showing the displacements of each atom from the mean plane of the 24-atom porphyrin macrocycle in units of 0.01 Å. The zinc atom is displaced by 0.4365 (4) Å from the 24-atom porphyrin mean plane (P_C_). This Zn^__^P_C_ distance is close to those of [Zn(OEP)(dabco)] (Konarev *et al.*, 2009 ▸) and [Zn(TPP)(pyridine)] which are 0.572 and 0.418 Å, respectively (Furuta *et al.*, 2002 ▸). The porphyrin core presents a major *saddle* and a moderate *ruffling* distortion (Scheidt & Lee, 1987 ▸). The *saddle* deformation is due to the displacement of the pyrrole rings alternately above and below the mean porphyrin macrocycle so that the pyrrole nitro­gen atoms are out of the mean plane. The *ruffling* distortion is indicated by the high values of the displacement of the meso-carbon atoms above and below the porphyrin mean plane.

## Supra­molecular features   

**Figure 3:**
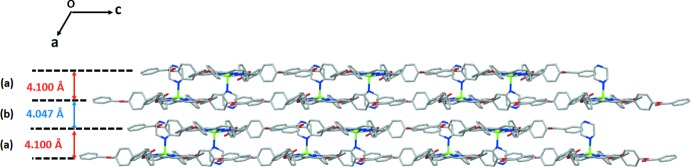
The packing of (I) viewed along [010] showing the two-dimensional superstructure formed by pairs of layers.

**Figure 4:**
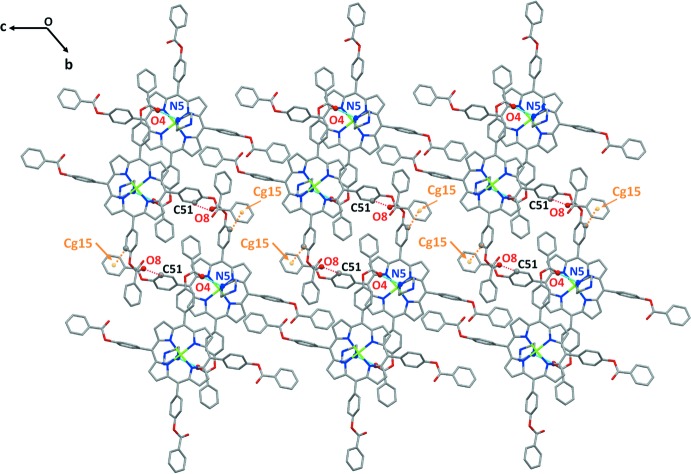
The packing of (I) viewed along [100] showing the inter­molecular inter­actions between two layers and between two pairs of layers.

In the crystal of compound (I), the [Zn(TPBP)(pipz)] mol­ecules are linked together in such way to make a pair of layers, parallel to (100), which are parallel to other pairs. The overall supra­molecular architecture in (I) is two-dimensional (Fig. 3 ▸). The distance between two layers is 4.100 Å while the pairs of layers are spaced apart by 4.047 Å. Within a layer, the linkage of the [Zn(TPBP)(pipz)] mol­ecules is accomplished by C^__^H⋯π inter­actions between the carbon atom C56 of a phenyl ring of one TPBP porphyrinate and the centroid *Cg*10 of a phenyl ring of an adjacent TPBP species [C56^__^H56⋯*Cg*10 = 3.623 (3) Å; Table 1 ▸). Each pair of layers is stabilized by N^__^H⋯O hydrogen bonds, C^__^H⋯O and C^__^H⋯π inter­molecular inter­actions (Table 1 ▸, Figs. 4 ▸ and 5 ▸). The values of these bond lengths are 2.904 (3) Å for N5^__^H5⋯O4, 3.284 (4) Å for C51^__^H51⋯O8, 3.566 (3) Å for C64^__^H64⋯*Cg*15 and 3.672 (3) Å for C69^__^H69⋯*Cg*11 (Table 1 ▸, Fig. 4 ▸). The parallel pairs of layers are sustained by the N6^__^H6⋯N1 weak hydrogen bond [3.434 (4) Å], the C63^__^H62⋯O6 [3.339 (4) Å], the C39^__^H39⋯*Cg*3 [3.392 (2) Å], the C48^__^H48⋯*Cg*12 [3.755 (3) Å] and the C49^__^H49⋯*Cg*17 [3.804 (3) Å] inter­molecular inter­actions.

## Synthesis and crystallization   

### Synthesis of the starting materials   

The {5,10,15,20-tetra­kis­[4-(benzo­yloxy)phen­yl]porphyrin} (H_2_TPBP) and the [Zn(TPBP)] starting complex were synthesized using modified reported methods (Adler *et al.*, 1967 ▸; Oberda *et al.*, 2011 ▸).

### Synthesis of the Synthesis and crystallization of the title complex (I)   

To a solution of the [Zn(TPBP)] starting material (100 mg, 0.086 mmol) in chloro­form (5 mL) was added an excess of piperazine hexa­hydrate (200 mg, 1.0297 mmol). The reaction mixture was stirred at room temperature for 2 h. Crystals of the title complex were obtained by diffusion of hexa­nes through the chloro­form solution. UV/Vis (CHCl_3_/solid), λ_max_: 430/445, 563/568, 603/609.

## UV-visible spectra   

**Figure 6:**
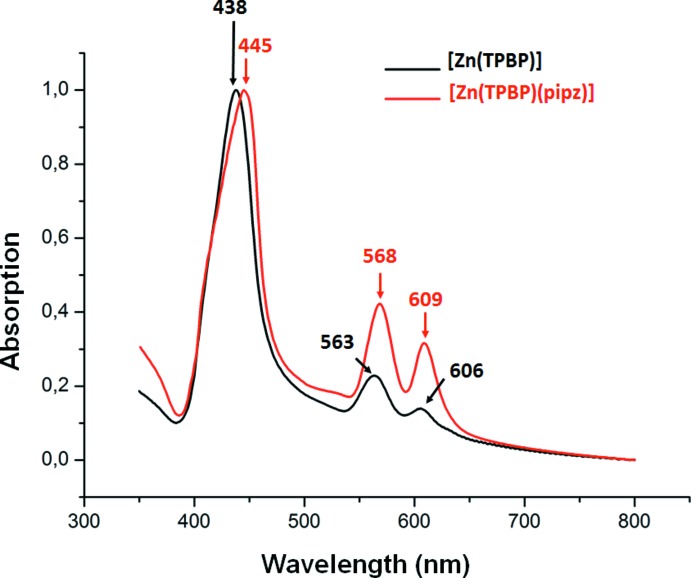
Solid UV–visible spectra of the [Zn(TPBP)] starting material (black) and complex (I) (red).

The UV–visible spectra (CHCl_3_ solution/solid state) were recorded on a WinASPECT PLUS (validation for SPECORD PLUS version 4.2) scanning spectrophotometer. Fig. 6 ▸ illustrates the electronic spectra of the solid [Zn(TPBP)] complex, used as starting material, and complex (I) which shows that the Soret and Q band of the latter species is red-shifted compared to those of the starting material. Thus, the λ_max_ (in nm) values of the Soret and Q bands of [Zn(TPBP)] and (I) are 438/445, 563/568 and 606/609 respectively. By the other hand, for (I), the values of theses absorption bands in chloro­form are blue-shifted compared to those in the solid state. In fact the λ_max_ (in nm) values are 430/445 for the Soret band and 563/568 and 603/609 for the Q bands.

## Refinement   

Crystal data, data collection and structure refinement details are summarized in Table 2 ▸. In the final refinement of (I) four reflections, *viz*. (121), (1
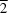
1), (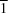
24) and (700), were omitted owing to poor agreements between observed and calculated intensities.

**Figure 7:**
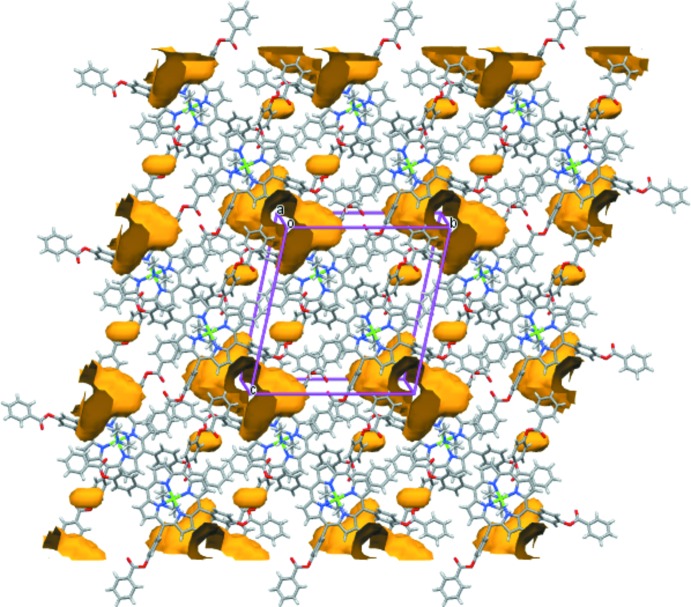
Packing diagram of (I) showing the voids in the structure represented in orange. Voids were calculated for a ball radius of 1.2 Å and a grid of 0.7 Å.

All H atoms attached to C atoms were fixed geometrically and treated as riding with C—H = 0.99 Å (methyl­ene) and 0.95 Å (aromatic) with *U*_iso_(H) = 1.2*U*_eq_(C). The two H atoms of the piperazine axial ligand were found in the difference Fourier map and the hydrogen atom of the nitro­gen N5 of the piperazine ligand coordinating to the Zn^2+^ atom was freely refined while the hydrogen atom of the second nitro­gen (N6) of the piperazine ligand was refined with fixed isotropic displacement parameters with *U*_iso_ =1.2*U*_eq_(N6). The bond length N5^__^H5 of the piperzine axial ligand was restrained to ensure proper geometry using DFIX instruction of *SHELXL2014* (Sheldrick, 2015 ▸). The anisotropic displacement ellipsoids of the carbon and nitro­gen atoms of the same piperazine ligand were very elongated, which indicates static disorder. For these atoms, a SIMU restraint was applied (McArdle, 1995 ▸; Sheldrick, 2008 ▸). An unknown *n*-hexane and water disordered mol­ecules were difficult to model, therefore solvent contributions to the scattering have been removed using the SQUEEZE procedure (Spek, 2015 ▸) in *PLATON* (Spek, 2009 ▸). SQUEEZE calculated a void volume of approximately 530 Å^3^ occupied by 60 electrons per unit cell, which points to the presence of approximately a half *n*-hexane and a water mol­ecule per formula unit. Fig. 7 ▸ shows the positions of the voids within the unit cell.

## Supplementary Material

Crystal structure: contains datablock(s) I. DOI: 10.1107/S2056989016009269/lh5815sup1.cif

Structure factors: contains datablock(s) I. DOI: 10.1107/S2056989016009269/lh5815Isup2.hkl

CCDC reference: 1483991

Additional supporting information: crystallographic information; 3D view; checkCIF report

## Figures and Tables

**Figure 5:**
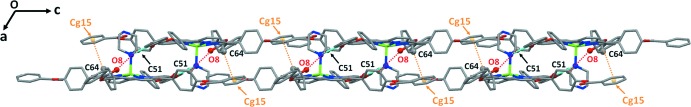
A drawing of (I) viewed along the [100] direction showing the inter­molecular inter­actions between two layers and between two pairs of layers.

**Table 1:** Hydrogen-bond geometry (Å, °) *Cg*3 is the centroid of the N3/C11–C14 pyrrole ring. *Cg*10, *Cg*11, *Cg*12, *Cg*15 and *Cg*17 are the centroids of the C21–C26, C28–C33, C34–C39, C54–59 and C67–C72 phenyl rings respectively.

*D*—H⋯*A*	*D*—H	H⋯*A*	*D*⋯*A*	*D*—H⋯*A*
N5—H5⋯O4^i^	0.80 (3)	2.15 (3)	2.904 (3)	158 (3)
N6—H6⋯N1^ii^	0.96 (2)	2.57 (3)	3.434 (4)	151 (3)
C51—H51⋯O8^iii^	0.95	2.47	3.284 (4)	144
C62—H62⋯O6^iv^	0.95	2.45	3.339 (4)	155
C39—H39⋯*Cg*3^v^	0.95	2.81	3.392 (2)	120
C48—H48⋯*Cg*12^v^	0.95	2.88	3.755 (3)	153
C49—H49⋯*Cg*17^iv^	0.95	2.90	3.804 (3)	160
C56—H56⋯*Cg*10^vi^	0.95	2.78	3.623 (3)	147
C64—H64⋯*Cg*15^iii^	0.95	2.64	3.566 (3)	164
C69—H69⋯*Cg*11^vii^	0.95	2.95	3.672 (3)	134

Symmetry codes: (i) 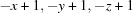; (ii) 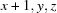; (iii) 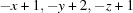; (iv) 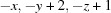; (v) 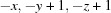; (vi) 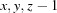; (vii) 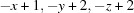.

**Table 2:** Experimental details

Crystal data	
Chemical formula	[Zn(C_72_H_44_N_4_O_8_)(C_4_H_10_N_2_)]
*M*_r_	1244.62
Crystal system, space group	Triclinic, *P* 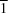
Temperature (K)	150
*a*, *b*, *c* (Å)	8.4332 (8), 20.1895 (17), 21.0104 (19)
α, β, γ (°)	102.338 (3), 100.996 (3), 98.412 (3)
*V* (Å^3^)	3364.6 (5)
*Z*	2
Radiation type	Mo *K*α
μ (mm^−1^)	0.43
Crystal size (mm)	0.30 × 0.20 × 0.16

Data collection	
Diffractometer	D8 VENTURE Bruker AXS
Absorption correction	Multi-scan (*SADABS*; Bruker, 2015 ▸)
*T*_min_, *T*_max_	0.684, 0.746
No. of measured, independent and observed [*I* > 2σ(*I*)] reflections	60504, 13198, 11791
*R*_int_	0.028
(sin θ/λ)_max_ (Å^−1^)	0.617

Refinement	
*R*[*F* ^2^ > 2σ(*F* ^2^)], *wR*(*F* ^2^), *S*	0.043, 0.119, 1.05
No. of reflections	13198
No. of parameters	827
No. of restraints	43
H-atom treatment	H atoms treated by a mixture of independent and constrained refinement
Δρ_max_, Δρ_min_ (e Å^−3^)	0.63, −0.54

Computer programs: *APEX3* and *SAINT* (Bruker, 2015 ▸), *SIR2004* (Burla *et al.*, 2005 ▸), *SHELXL2015* (Sheldrick, 2015 ▸), *ORTEPIII* (Burnett & Johnson, 1996 ▸), *ORTEP-3 for Windows* and *WinGX* publication routines (Farrugia, 2012 ▸).

## supplementary crystallographic information

### Crystal data

**Table:**

[Zn(C_72_H_44_N_4_O_8_)(C_4_H_10_N_2_)]	*Z* = 2
*M**_r_* = 1244.62	*F*(000) = 1292
Triclinic, *P*1	*D*_x_ = 1.229 Mg m^−^^3^
*a* = 8.4332 (8) Å	Mo *K*α radiation, λ = 0.71073 Å
*b* = 20.1895 (17) Å	Cell parameters from 9221 reflections
*c* = 21.0104 (19) Å	θ = 2.5–27.5°
α = 102.338 (3)°	µ = 0.43 mm^−^^1^
β = 100.996 (3)°	*T* = 150 K
γ = 98.412 (3)°	Prism, blue
*V* = 3364.6 (5) Å^3^	0.30 × 0.20 × 0.16 mm

### Data collection

**Table:**

D8 VENTURE Bruker AXS diffractometer	11791 reflections with *I* > 2σ(*I*)
rotation images scans	*R*_int_ = 0.028
Absorption correction: multi-scan (SADABS; Bruker, 2015)	θ_max_ = 26.0°, θ_min_ = 2.9°
*T*_min_ = 0.684, *T*_max_ = 0.746	*h* = −10→10
60504 measured reflections	*k* = −23→24
13198 independent reflections	*l* = −25→25

### Refinement

**Table:**

Refinement on *F*^2^	43 restraints
Least-squares matrix: full	Hydrogen site location: mixed
*R*[*F*^2^ > 2σ(*F*^2^)] = 0.043	H atoms treated by a mixture of independent and constrained refinement
*wR*(*F*^2^) = 0.119	*w* = 1/[σ^2^(*F*_o_^2^) + (0.0565*P*)^2^ + 3.3556*P*] where *P* = (*F*_o_^2^ + 2*F*_c_^2^)/3
*S* = 1.05	(Δ/σ)_max_ = 0.002
13198 reflections	Δρ_max_ = 0.63 e Å^−^^3^
827 parameters	Δρ_min_ = −0.54 e Å^−^^3^

### Special details

**Table:**

Geometry. All esds (except the esd in the dihedral angle between two l.s. planes) are estimated using the full covariance matrix. The cell esds are taken into account individually in the estimation of esds in distances, angles and torsion angles; correlations between esds in cell parameters are only used when they are defined by crystal symmetry. An approximate (isotropic) treatment of cell esds is used for estimating esds involving l.s. planes.

### Fractional atomic coordinates and isotropic or equivalent isotropic displacement parameters (Å^2^)

**Table:**

	*x*	*y*	*z*	*U*_iso_*/*U*_eq_	
Zn	0.31335 (3)	0.69459 (2)	0.65779 (2)	0.01812 (7)	
N1	0.2782 (2)	0.75429 (8)	0.74622 (8)	0.0211 (3)	
N2	0.2627 (2)	0.60823 (8)	0.69420 (8)	0.0206 (3)	
N3	0.2614 (2)	0.62837 (8)	0.56245 (8)	0.0197 (3)	
N4	0.2644 (2)	0.77404 (8)	0.61332 (8)	0.0194 (3)	
N5	0.5751 (2)	0.71283 (11)	0.67848 (10)	0.0301 (4)	
H5	0.602 (4)	0.7385 (17)	0.6565 (17)	0.056 (9)*	
N6	0.8803 (3)	0.6862 (3)	0.74598 (19)	0.0930 (13)	
H6	0.997 (2)	0.692 (2)	0.754 (2)	0.112*	
O1	0.2385 (2)	0.60093 (8)	1.05857 (7)	0.0349 (4)	
O2	0.2374 (3)	0.49041 (10)	1.01050 (9)	0.0596 (6)	
O3	0.2188 (2)	0.22666 (7)	0.51518 (8)	0.0293 (3)	
O4	0.2934 (4)	0.22820 (11)	0.41963 (11)	0.0704 (8)	
O5	0.2117 (2)	0.75993 (8)	0.23573 (7)	0.0282 (3)	
O6	0.1746 (3)	0.86806 (10)	0.27232 (9)	0.0504 (5)	
O7	0.2304 (2)	1.14575 (8)	0.80769 (9)	0.0390 (4)	
O8	0.3722 (3)	1.17651 (10)	0.73573 (13)	0.0715 (8)	
C1	0.2767 (3)	0.82347 (10)	0.76077 (10)	0.0230 (4)	
C2	0.2803 (3)	0.84814 (11)	0.83101 (11)	0.0319 (5)	
H2	0.2803	0.8942	0.8538	0.038*	
C3	0.2836 (3)	0.79322 (11)	0.85815 (11)	0.0309 (5)	
H3	0.2872	0.7934	0.9037	0.037*	
C4	0.2804 (3)	0.73428 (11)	0.80492 (10)	0.0228 (4)	
C5	0.2646 (2)	0.66604 (11)	0.81071 (10)	0.0215 (4)	
C6	0.2465 (2)	0.60762 (10)	0.75811 (10)	0.0204 (4)	
C7	0.2050 (3)	0.53744 (11)	0.76309 (10)	0.0257 (4)	
H7	0.1861	0.5230	0.8016	0.031*	
C8	0.1981 (3)	0.49601 (11)	0.70258 (11)	0.0262 (4)	
H8	0.1720	0.4469	0.6904	0.031*	
C9	0.2378 (2)	0.54025 (10)	0.65971 (10)	0.0208 (4)	
C10	0.2509 (2)	0.51649 (10)	0.59287 (10)	0.0211 (4)	
C11	0.2661 (2)	0.55911 (10)	0.54829 (10)	0.0206 (4)	
C12	0.2819 (3)	0.53651 (11)	0.48024 (10)	0.0252 (4)	
H12	0.2920	0.4915	0.4585	0.030*	
C13	0.2798 (3)	0.59145 (11)	0.45319 (10)	0.0251 (4)	
H13	0.2860	0.5920	0.4086	0.030*	
C14	0.2662 (2)	0.64918 (10)	0.50448 (9)	0.0204 (4)	
C15	0.2564 (2)	0.71571 (10)	0.49630 (9)	0.0198 (4)	
C16	0.2521 (2)	0.77297 (10)	0.54688 (9)	0.0200 (4)	
C17	0.2310 (3)	0.83986 (11)	0.53666 (10)	0.0266 (4)	
H17	0.2174	0.8526	0.4953	0.032*	
C18	0.2342 (3)	0.88089 (11)	0.59688 (11)	0.0288 (5)	
H18	0.2246	0.9281	0.6059	0.035*	
C19	0.2547 (2)	0.83975 (10)	0.64507 (10)	0.0220 (4)	
C20	0.2640 (3)	0.86390 (10)	0.71389 (10)	0.0234 (4)	
C21	0.2579 (3)	0.65248 (10)	0.87802 (10)	0.0226 (4)	
C22	0.1093 (3)	0.63974 (14)	0.89588 (11)	0.0364 (5)	
H22	0.0111	0.6433	0.8671	0.044*	
C23	0.1023 (3)	0.62159 (14)	0.95589 (12)	0.0379 (6)	
H23	−0.0002	0.6123	0.9680	0.045*	
C24	0.2450 (3)	0.61738 (11)	0.99694 (10)	0.0290 (5)	
C25	0.3944 (3)	0.63187 (16)	0.98167 (13)	0.0460 (7)	
H25	0.4926	0.6298	1.0115	0.055*	
C26	0.4004 (3)	0.64969 (16)	0.92169 (13)	0.0417 (6)	
H26	0.5037	0.6601	0.9106	0.050*	
C27	0.2413 (3)	0.53406 (12)	1.05929 (11)	0.0334 (5)	
C28	0.2507 (3)	0.52300 (11)	1.12744 (10)	0.0274 (4)	
C29	0.2114 (3)	0.56867 (13)	1.17817 (11)	0.0352 (5)	
H29	0.1766	0.6096	1.1708	0.042*	
C30	0.2235 (4)	0.55373 (15)	1.24035 (12)	0.0439 (6)	
H30	0.1964	0.5848	1.2756	0.053*	
C31	0.2737 (3)	0.49517 (14)	1.25143 (12)	0.0402 (6)	
H31	0.2793	0.4852	1.2939	0.048*	
C32	0.3166 (4)	0.45029 (14)	1.20094 (14)	0.0474 (7)	
H32	0.3549	0.4102	1.2091	0.057*	
C33	0.3039 (4)	0.46368 (13)	1.13876 (13)	0.0409 (6)	
H33	0.3315	0.4325	1.1037	0.049*	
C34	0.2463 (2)	0.44089 (10)	0.56896 (10)	0.0213 (4)	
C35	0.3511 (3)	0.40835 (11)	0.60647 (10)	0.0259 (4)	
H35	0.4294	0.4352	0.6456	0.031*	
C36	0.3427 (3)	0.33756 (11)	0.58756 (11)	0.0269 (4)	
H36	0.4134	0.3159	0.6137	0.032*	
C37	0.2300 (3)	0.29906 (10)	0.53018 (10)	0.0235 (4)	
C38	0.1260 (2)	0.32938 (10)	0.49152 (10)	0.0229 (4)	
H38	0.0499	0.3023	0.4520	0.028*	
C39	0.1346 (2)	0.40020 (10)	0.51142 (10)	0.0224 (4)	
H39	0.0628	0.4214	0.4852	0.027*	
C40	0.2577 (3)	0.19646 (11)	0.45797 (11)	0.0280 (4)	
C41	0.2568 (3)	0.12180 (11)	0.44955 (12)	0.0327 (5)	
C42	0.2004 (6)	0.08583 (16)	0.4910 (2)	0.0737 (11)	
H42	0.1603	0.1080	0.5276	0.088*	
C43	0.2028 (9)	0.0150 (2)	0.4782 (3)	0.123 (2)	
H43	0.1636	−0.0113	0.5065	0.148*	
C44	0.2613 (7)	−0.01704 (18)	0.4251 (2)	0.0913 (15)	
H44	0.2608	−0.0652	0.4168	0.110*	
C45	0.3189 (4)	0.01918 (15)	0.38517 (17)	0.0591 (8)	
H45	0.3621	−0.0027	0.3494	0.071*	
C46	0.3148 (3)	0.08787 (14)	0.39637 (14)	0.0445 (6)	
H46	0.3524	0.1132	0.3671	0.053*	
C47	0.2435 (2)	0.72691 (10)	0.42727 (9)	0.0202 (4)	
C48	0.1063 (3)	0.69477 (11)	0.37587 (10)	0.0257 (4)	
H48	0.0202	0.6641	0.3840	0.031*	
C49	0.0934 (3)	0.70708 (11)	0.31222 (10)	0.0278 (4)	
H49	−0.0015	0.6858	0.2774	0.033*	
C50	0.2210 (3)	0.75070 (10)	0.30097 (10)	0.0232 (4)	
C51	0.3586 (3)	0.78352 (11)	0.35088 (10)	0.0256 (4)	
H51	0.4451	0.8135	0.3422	0.031*	
C52	0.3682 (3)	0.77189 (11)	0.41419 (10)	0.0240 (4)	
H52	0.4615	0.7950	0.4492	0.029*	
C53	0.1959 (3)	0.82397 (12)	0.22857 (10)	0.0289 (5)	
C54	0.2081 (3)	0.83278 (12)	0.16118 (11)	0.0305 (5)	
C55	0.2608 (4)	0.78526 (13)	0.11602 (12)	0.0428 (6)	
H55	0.2843	0.7436	0.1262	0.051*	
C56	0.2791 (5)	0.79915 (16)	0.05560 (15)	0.0667 (10)	
H56	0.3154	0.7669	0.0242	0.080*	
C57	0.2445 (6)	0.85958 (18)	0.04113 (15)	0.0726 (11)	
H57	0.2590	0.8691	0.0000	0.087*	
C58	0.1895 (5)	0.90623 (18)	0.08526 (15)	0.0632 (9)	
H58	0.1641	0.9474	0.0744	0.076*	
C59	0.1714 (4)	0.89299 (15)	0.14549 (13)	0.0454 (6)	
H59	0.1337	0.9252	0.1763	0.055*	
C60	0.2566 (3)	0.93847 (10)	0.73884 (10)	0.0240 (4)	
C61	0.1139 (3)	0.96266 (11)	0.71824 (11)	0.0304 (5)	
H61	0.0203	0.9314	0.6890	0.036*	
C62	0.1066 (3)	1.03198 (12)	0.73989 (12)	0.0328 (5)	
H62	0.0096	1.0484	0.7251	0.039*	
C63	0.2414 (3)	1.07619 (11)	0.78290 (12)	0.0325 (5)	
C64	0.3835 (3)	1.05388 (12)	0.80529 (13)	0.0392 (6)	
H64	0.4751	1.0853	0.8357	0.047*	
C65	0.3910 (3)	0.98463 (12)	0.78275 (12)	0.0346 (5)	
H65	0.4890	0.9688	0.7975	0.042*	
C66	0.3034 (3)	1.19226 (12)	0.77925 (13)	0.0366 (5)	
C67	0.2864 (3)	1.26376 (11)	0.80969 (12)	0.0314 (5)	
C68	0.3596 (3)	1.31723 (13)	0.78607 (14)	0.0403 (6)	
H68	0.4184	1.3074	0.7520	0.048*	
C69	0.3464 (3)	1.38420 (13)	0.81222 (14)	0.0429 (6)	
H69	0.3953	1.4205	0.7957	0.051*	
C70	0.2631 (4)	1.39908 (12)	0.86208 (13)	0.0431 (6)	
H70	0.2565	1.4456	0.8804	0.052*	
C71	0.1888 (4)	1.34650 (13)	0.88561 (13)	0.0436 (6)	
H71	0.1299	1.3568	0.9196	0.052*	
C72	0.2004 (3)	1.27843 (12)	0.85936 (12)	0.0340 (5)	
H72	0.1496	1.2422	0.8754	0.041*	
C73	0.6390 (3)	0.64968 (16)	0.65693 (17)	0.0507 (7)	
H73A	0.6017	0.6325	0.6077	0.061*	
H73B	0.5919	0.6136	0.6771	0.061*	
C74	0.8263 (3)	0.6608 (2)	0.67635 (19)	0.0639 (9)	
H74A	0.8623	0.6166	0.6620	0.077*	
H74B	0.8749	0.6943	0.6537	0.077*	
C75	0.8406 (4)	0.7520 (3)	0.7659 (2)	0.0875 (13)	
H75A	0.8896	0.7837	0.7417	0.105*	
H75B	0.8865	0.7714	0.8145	0.105*	
C76	0.6495 (4)	0.7452 (2)	0.74997 (16)	0.0702 (10)	
H76A	0.6023	0.7170	0.7775	0.084*	
H76B	0.6222	0.7917	0.7620	0.084*	

### Atomic displacement parameters (Å^2^)

**Table:**

	*U*^11^	*U*^22^	*U*^33^	*U*^12^	*U*^13^	*U*^23^
Zn	0.02219 (12)	0.01640 (12)	0.01721 (12)	0.00479 (8)	0.00457 (8)	0.00655 (8)
N1	0.0273 (9)	0.0183 (8)	0.0195 (8)	0.0045 (7)	0.0062 (7)	0.0074 (6)
N2	0.0251 (8)	0.0202 (8)	0.0187 (8)	0.0057 (7)	0.0077 (6)	0.0064 (6)
N3	0.0253 (8)	0.0167 (8)	0.0193 (8)	0.0053 (6)	0.0061 (6)	0.0075 (6)
N4	0.0246 (8)	0.0162 (8)	0.0180 (8)	0.0049 (6)	0.0038 (6)	0.0058 (6)
N5	0.0250 (9)	0.0386 (11)	0.0312 (10)	0.0037 (8)	0.0063 (7)	0.0200 (9)
N6	0.0299 (14)	0.187 (4)	0.087 (2)	0.0221 (19)	0.0123 (14)	0.087 (3)
O1	0.0634 (11)	0.0264 (8)	0.0203 (7)	0.0121 (7)	0.0129 (7)	0.0126 (6)
O2	0.125 (2)	0.0288 (9)	0.0314 (10)	0.0182 (11)	0.0311 (11)	0.0086 (8)
O3	0.0429 (9)	0.0174 (7)	0.0303 (8)	0.0061 (6)	0.0134 (7)	0.0073 (6)
O4	0.142 (2)	0.0429 (12)	0.0652 (14)	0.0478 (13)	0.0744 (15)	0.0320 (11)
O5	0.0431 (9)	0.0264 (8)	0.0173 (7)	0.0075 (6)	0.0096 (6)	0.0076 (6)
O6	0.0925 (15)	0.0484 (11)	0.0311 (9)	0.0468 (11)	0.0282 (10)	0.0194 (8)
O7	0.0599 (11)	0.0186 (8)	0.0464 (10)	0.0098 (7)	0.0293 (9)	0.0085 (7)
O8	0.113 (2)	0.0356 (11)	0.1010 (18)	0.0283 (12)	0.0867 (17)	0.0282 (11)
C1	0.0288 (10)	0.0186 (9)	0.0211 (10)	0.0043 (8)	0.0053 (8)	0.0043 (8)
C2	0.0510 (14)	0.0212 (10)	0.0223 (10)	0.0068 (9)	0.0089 (10)	0.0024 (8)
C3	0.0491 (14)	0.0250 (11)	0.0189 (10)	0.0076 (10)	0.0083 (9)	0.0057 (8)
C4	0.0273 (10)	0.0229 (10)	0.0191 (9)	0.0049 (8)	0.0055 (8)	0.0072 (8)
C5	0.0225 (10)	0.0251 (10)	0.0197 (9)	0.0063 (8)	0.0059 (7)	0.0095 (8)
C6	0.0217 (9)	0.0223 (10)	0.0211 (9)	0.0060 (8)	0.0073 (7)	0.0101 (8)
C7	0.0351 (11)	0.0218 (10)	0.0255 (10)	0.0060 (8)	0.0121 (9)	0.0121 (8)
C8	0.0345 (11)	0.0188 (10)	0.0285 (10)	0.0054 (8)	0.0102 (9)	0.0100 (8)
C9	0.0243 (10)	0.0168 (9)	0.0238 (10)	0.0057 (7)	0.0074 (8)	0.0075 (8)
C10	0.0225 (9)	0.0182 (9)	0.0246 (10)	0.0059 (7)	0.0072 (8)	0.0066 (8)
C11	0.0232 (9)	0.0190 (9)	0.0212 (9)	0.0063 (7)	0.0072 (7)	0.0053 (7)
C12	0.0340 (11)	0.0194 (10)	0.0260 (10)	0.0079 (8)	0.0140 (9)	0.0054 (8)
C13	0.0346 (11)	0.0228 (10)	0.0223 (10)	0.0074 (8)	0.0144 (8)	0.0070 (8)
C14	0.0226 (9)	0.0211 (10)	0.0187 (9)	0.0041 (7)	0.0068 (7)	0.0059 (7)
C15	0.0211 (9)	0.0213 (10)	0.0185 (9)	0.0029 (7)	0.0054 (7)	0.0083 (7)
C16	0.0224 (9)	0.0197 (9)	0.0197 (9)	0.0049 (7)	0.0041 (7)	0.0087 (7)
C17	0.0382 (12)	0.0229 (10)	0.0225 (10)	0.0105 (9)	0.0069 (9)	0.0108 (8)
C18	0.0445 (13)	0.0191 (10)	0.0264 (10)	0.0118 (9)	0.0083 (9)	0.0094 (8)
C19	0.0276 (10)	0.0169 (9)	0.0213 (10)	0.0052 (8)	0.0040 (8)	0.0051 (7)
C20	0.0281 (10)	0.0183 (10)	0.0226 (10)	0.0047 (8)	0.0038 (8)	0.0041 (8)
C21	0.0310 (11)	0.0197 (9)	0.0192 (9)	0.0056 (8)	0.0071 (8)	0.0077 (8)
C22	0.0295 (12)	0.0573 (16)	0.0253 (11)	0.0065 (11)	0.0043 (9)	0.0192 (11)
C23	0.0377 (13)	0.0534 (15)	0.0268 (11)	0.0044 (11)	0.0127 (10)	0.0175 (11)
C24	0.0492 (13)	0.0226 (10)	0.0176 (9)	0.0068 (9)	0.0086 (9)	0.0094 (8)
C25	0.0402 (14)	0.0713 (19)	0.0387 (14)	0.0205 (13)	0.0070 (11)	0.0348 (14)
C26	0.0321 (12)	0.0675 (18)	0.0386 (13)	0.0183 (12)	0.0133 (10)	0.0309 (13)
C27	0.0529 (15)	0.0245 (11)	0.0262 (11)	0.0075 (10)	0.0133 (10)	0.0101 (9)
C28	0.0342 (11)	0.0259 (11)	0.0246 (10)	0.0052 (9)	0.0068 (9)	0.0119 (8)
C29	0.0499 (14)	0.0353 (13)	0.0296 (12)	0.0208 (11)	0.0127 (10)	0.0161 (10)
C30	0.0678 (18)	0.0473 (15)	0.0268 (12)	0.0234 (13)	0.0192 (12)	0.0152 (11)
C31	0.0515 (15)	0.0436 (14)	0.0281 (12)	0.0071 (12)	0.0042 (11)	0.0201 (11)
C32	0.0670 (18)	0.0365 (14)	0.0441 (15)	0.0163 (13)	0.0062 (13)	0.0233 (12)
C33	0.0658 (17)	0.0263 (12)	0.0359 (13)	0.0152 (11)	0.0149 (12)	0.0120 (10)
C34	0.0247 (10)	0.0187 (9)	0.0236 (10)	0.0052 (8)	0.0101 (8)	0.0071 (8)
C35	0.0269 (10)	0.0236 (10)	0.0254 (10)	0.0067 (8)	0.0031 (8)	0.0036 (8)
C36	0.0306 (11)	0.0242 (10)	0.0280 (10)	0.0109 (8)	0.0056 (9)	0.0079 (8)
C37	0.0291 (10)	0.0170 (9)	0.0276 (10)	0.0055 (8)	0.0127 (8)	0.0065 (8)
C38	0.0244 (10)	0.0219 (10)	0.0210 (9)	0.0004 (8)	0.0063 (8)	0.0039 (8)
C39	0.0239 (10)	0.0240 (10)	0.0235 (10)	0.0073 (8)	0.0083 (8)	0.0107 (8)
C40	0.0320 (11)	0.0251 (11)	0.0283 (11)	0.0075 (9)	0.0081 (9)	0.0069 (9)
C41	0.0325 (12)	0.0216 (11)	0.0393 (13)	0.0048 (9)	0.0024 (10)	0.0031 (9)
C42	0.129 (3)	0.0330 (16)	0.085 (2)	0.0285 (18)	0.066 (2)	0.0252 (16)
C43	0.251 (7)	0.038 (2)	0.126 (4)	0.044 (3)	0.118 (5)	0.041 (2)
C44	0.158 (4)	0.0319 (17)	0.100 (3)	0.036 (2)	0.057 (3)	0.0154 (19)
C45	0.072 (2)	0.0359 (15)	0.064 (2)	0.0163 (14)	0.0177 (16)	−0.0066 (14)
C46	0.0512 (16)	0.0350 (14)	0.0434 (14)	0.0151 (12)	0.0075 (12)	−0.0003 (11)
C47	0.0261 (10)	0.0186 (9)	0.0191 (9)	0.0074 (8)	0.0081 (8)	0.0071 (7)
C48	0.0286 (11)	0.0256 (10)	0.0222 (10)	−0.0006 (8)	0.0085 (8)	0.0059 (8)
C49	0.0318 (11)	0.0292 (11)	0.0191 (10)	0.0023 (9)	0.0036 (8)	0.0037 (8)
C50	0.0359 (11)	0.0218 (10)	0.0168 (9)	0.0116 (8)	0.0101 (8)	0.0073 (7)
C51	0.0273 (10)	0.0277 (11)	0.0264 (10)	0.0052 (8)	0.0101 (8)	0.0133 (8)
C52	0.0240 (10)	0.0256 (10)	0.0228 (10)	0.0041 (8)	0.0035 (8)	0.0093 (8)
C53	0.0361 (12)	0.0341 (12)	0.0223 (10)	0.0142 (9)	0.0092 (9)	0.0121 (9)
C54	0.0364 (12)	0.0334 (12)	0.0218 (10)	0.0030 (9)	0.0067 (9)	0.0098 (9)
C55	0.0736 (19)	0.0261 (12)	0.0275 (12)	−0.0007 (12)	0.0199 (12)	0.0037 (9)
C56	0.131 (3)	0.0374 (16)	0.0344 (15)	0.0052 (17)	0.0406 (18)	0.0028 (12)
C57	0.140 (4)	0.0543 (19)	0.0299 (14)	0.011 (2)	0.0318 (18)	0.0190 (13)
C58	0.109 (3)	0.0567 (19)	0.0386 (15)	0.0280 (19)	0.0227 (17)	0.0297 (14)
C59	0.0637 (18)	0.0512 (16)	0.0337 (13)	0.0245 (14)	0.0170 (12)	0.0224 (12)
C60	0.0345 (11)	0.0174 (10)	0.0214 (10)	0.0063 (8)	0.0070 (8)	0.0058 (8)
C61	0.0378 (12)	0.0231 (11)	0.0277 (11)	0.0079 (9)	0.0047 (9)	0.0018 (9)
C62	0.0418 (13)	0.0270 (11)	0.0330 (12)	0.0149 (10)	0.0107 (10)	0.0070 (9)
C63	0.0510 (14)	0.0172 (10)	0.0344 (12)	0.0094 (9)	0.0201 (10)	0.0059 (9)
C64	0.0418 (14)	0.0210 (11)	0.0474 (14)	−0.0012 (10)	0.0055 (11)	0.0015 (10)
C65	0.0357 (12)	0.0232 (11)	0.0409 (13)	0.0054 (9)	0.0028 (10)	0.0047 (10)
C66	0.0428 (13)	0.0264 (12)	0.0482 (14)	0.0080 (10)	0.0248 (11)	0.0120 (10)
C67	0.0317 (11)	0.0204 (10)	0.0411 (13)	0.0052 (9)	0.0065 (10)	0.0072 (9)
C68	0.0373 (13)	0.0316 (13)	0.0561 (16)	0.0067 (10)	0.0138 (12)	0.0171 (11)
C69	0.0402 (14)	0.0244 (12)	0.0576 (16)	−0.0008 (10)	−0.0049 (12)	0.0155 (11)
C70	0.0583 (16)	0.0189 (11)	0.0414 (14)	0.0103 (11)	−0.0087 (12)	0.0009 (10)
C71	0.0610 (17)	0.0329 (13)	0.0353 (13)	0.0183 (12)	0.0070 (12)	0.0027 (10)
C72	0.0428 (13)	0.0235 (11)	0.0340 (12)	0.0046 (9)	0.0066 (10)	0.0065 (9)
C73	0.0308 (13)	0.0567 (17)	0.0788 (19)	0.0193 (12)	0.0164 (13)	0.0365 (15)
C74	0.0311 (14)	0.094 (2)	0.089 (2)	0.0219 (14)	0.0167 (14)	0.0610 (19)
C75	0.0387 (17)	0.146 (4)	0.056 (2)	−0.015 (2)	−0.0030 (15)	0.014 (2)
C76	0.0332 (15)	0.114 (3)	0.0484 (17)	−0.0048 (16)	−0.0007 (12)	0.0094 (18)

### Geometric parameters (Å, º)

**Table:**

Zn—N2	2.0697 (16)	C31—H31	0.9500
Zn—N4	2.0747 (16)	C32—C33	1.377 (4)
Zn—N3	2.0836 (16)	C32—H32	0.9500
Zn—N1	2.0856 (17)	C33—H33	0.9500
Zn—N5	2.1274 (19)	C34—C39	1.392 (3)
N1—C1	1.367 (3)	C34—C35	1.400 (3)
N1—C4	1.375 (2)	C35—C36	1.386 (3)
N2—C9	1.373 (3)	C35—H35	0.9500
N2—C6	1.378 (2)	C36—C37	1.381 (3)
N3—C11	1.374 (2)	C36—H36	0.9500
N3—C14	1.376 (2)	C37—C38	1.379 (3)
N4—C19	1.373 (2)	C38—C39	1.388 (3)
N4—C16	1.375 (2)	C38—H38	0.9500
N5—C73	1.472 (4)	C39—H39	0.9500
N5—C76	1.476 (4)	C40—C41	1.478 (3)
N5—H5	0.80 (3)	C41—C42	1.356 (4)
N6—C74	1.403 (5)	C41—C46	1.391 (4)
N6—C75	1.412 (6)	C42—C43	1.402 (5)
N6—H6	0.955 (19)	C42—H42	0.9500
O1—C27	1.357 (3)	C43—C44	1.380 (6)
O1—C24	1.413 (2)	C43—H43	0.9500
O2—C27	1.192 (3)	C44—C45	1.337 (5)
O3—C40	1.349 (3)	C44—H44	0.9500
O3—C37	1.413 (2)	C45—C46	1.363 (4)
O4—C40	1.186 (3)	C45—H45	0.9500
O5—C53	1.356 (3)	C46—H46	0.9500
O5—C50	1.412 (2)	C47—C48	1.389 (3)
O6—C53	1.196 (3)	C47—C52	1.394 (3)
O7—C66	1.352 (3)	C48—C49	1.399 (3)
O7—C63	1.413 (3)	C48—H48	0.9500
O8—C66	1.186 (3)	C49—C50	1.378 (3)
C1—C20	1.406 (3)	C49—H49	0.9500
C1—C2	1.446 (3)	C50—C51	1.378 (3)
C2—C3	1.352 (3)	C51—C52	1.389 (3)
C2—H2	0.9500	C51—H51	0.9500
C3—C4	1.442 (3)	C52—H52	0.9500
C3—H3	0.9500	C53—C54	1.485 (3)
C4—C5	1.398 (3)	C54—C55	1.384 (3)
C5—C6	1.401 (3)	C54—C59	1.387 (3)
C5—C21	1.506 (3)	C55—C56	1.388 (4)
C6—C7	1.440 (3)	C55—H55	0.9500
C7—C8	1.350 (3)	C56—C57	1.376 (5)
C7—H7	0.9500	C56—H56	0.9500
C8—C9	1.446 (3)	C57—C58	1.371 (5)
C8—H8	0.9500	C57—H57	0.9500
C9—C10	1.415 (3)	C58—C59	1.379 (4)
C10—C11	1.411 (3)	C58—H58	0.9500
C10—C34	1.494 (3)	C59—H59	0.9500
C11—C12	1.443 (3)	C60—C65	1.389 (3)
C12—C13	1.351 (3)	C60—C61	1.392 (3)
C12—H12	0.9500	C61—C62	1.390 (3)
C13—C14	1.443 (3)	C61—H61	0.9500
C13—H13	0.9500	C62—C63	1.368 (3)
C14—C15	1.402 (3)	C62—H62	0.9500
C15—C16	1.403 (3)	C63—C64	1.376 (4)
C15—C47	1.501 (3)	C64—C65	1.392 (3)
C16—C17	1.443 (3)	C64—H64	0.9500
C17—C18	1.349 (3)	C65—H65	0.9500
C17—H17	0.9500	C66—C67	1.488 (3)
C18—C19	1.442 (3)	C67—C72	1.386 (3)
C18—H18	0.9500	C67—C68	1.394 (3)
C19—C20	1.407 (3)	C68—C69	1.376 (4)
C20—C60	1.499 (3)	C68—H68	0.9500
C21—C22	1.379 (3)	C69—C70	1.375 (4)
C21—C26	1.382 (3)	C69—H69	0.9500
C22—C23	1.395 (3)	C70—C71	1.383 (4)
C22—H22	0.9500	C70—H70	0.9500
C23—C24	1.366 (3)	C71—C72	1.392 (3)
C23—H23	0.9500	C71—H71	0.9500
C24—C25	1.364 (4)	C72—H72	0.9500
C25—C26	1.390 (3)	C73—C74	1.524 (4)
C25—H25	0.9500	C73—H73A	0.9900
C26—H26	0.9500	C73—H73B	0.9900
C27—C28	1.485 (3)	C74—H74A	0.9900
C28—C29	1.379 (3)	C74—H74B	0.9900
C28—C33	1.389 (3)	C75—C76	1.562 (5)
C29—C30	1.391 (3)	C75—H75A	0.9900
C29—H29	0.9500	C75—H75B	0.9900
C30—C31	1.363 (4)	C76—H76A	0.9900
C30—H30	0.9500	C76—H76B	0.9900
C31—C32	1.382 (4)		
			
N2—Zn—N4	157.48 (7)	C36—C35—H35	119.4
N2—Zn—N3	87.83 (6)	C34—C35—H35	119.4
N4—Zn—N3	88.49 (6)	C37—C36—C35	118.99 (19)
N2—Zn—N1	88.20 (6)	C37—C36—H36	120.5
N4—Zn—N1	87.89 (6)	C35—C36—H36	120.5
N3—Zn—N1	160.46 (7)	C38—C37—C36	121.55 (19)
N2—Zn—N5	101.14 (7)	C38—C37—O3	121.41 (19)
N4—Zn—N5	101.38 (7)	C36—C37—O3	116.93 (18)
N3—Zn—N5	99.26 (7)	C37—C38—C39	118.86 (19)
N1—Zn—N5	100.27 (7)	C37—C38—H38	120.6
C1—N1—C4	106.42 (16)	C39—C38—H38	120.6
C1—N1—Zn	126.77 (13)	C38—C39—C34	121.44 (18)
C4—N1—Zn	125.96 (13)	C38—C39—H39	119.3
C9—N2—C6	106.21 (16)	C34—C39—H39	119.3
C9—N2—Zn	127.12 (13)	O4—C40—O3	122.2 (2)
C6—N2—Zn	126.67 (13)	O4—C40—C41	124.1 (2)
C11—N3—C14	106.69 (16)	O3—C40—C41	113.66 (19)
C11—N3—Zn	125.75 (13)	C42—C41—C46	119.6 (2)
C14—N3—Zn	124.90 (13)	C42—C41—C40	122.5 (2)
C19—N4—C16	106.66 (16)	C46—C41—C40	117.9 (2)
C19—N4—Zn	126.93 (13)	C41—C42—C43	118.1 (3)
C16—N4—Zn	126.10 (13)	C41—C42—H42	121.0
C73—N5—C76	109.9 (2)	C43—C42—H42	121.0
C73—N5—Zn	112.62 (16)	C44—C43—C42	120.8 (4)
C76—N5—Zn	111.88 (17)	C44—C43—H43	119.6
C73—N5—H5	107 (2)	C42—C43—H43	119.6
C76—N5—H5	109 (2)	C45—C44—C43	120.5 (3)
Zn—N5—H5	106 (2)	C45—C44—H44	119.7
C74—N6—C75	109.8 (3)	C43—C44—H44	119.7
C74—N6—H6	104 (3)	C44—C45—C46	119.2 (3)
C75—N6—H6	108 (3)	C44—C45—H45	120.4
C27—O1—C24	115.51 (17)	C46—C45—H45	120.4
C40—O3—C37	117.59 (16)	C45—C46—C41	121.7 (3)
C53—O5—C50	115.50 (16)	C45—C46—H46	119.2
C66—O7—C63	116.07 (18)	C41—C46—H46	119.2
N1—C1—C20	125.22 (18)	C48—C47—C52	118.63 (18)
N1—C1—C2	109.82 (17)	C48—C47—C15	121.26 (17)
C20—C1—C2	124.87 (19)	C52—C47—C15	120.07 (18)
C3—C2—C1	106.97 (19)	C47—C48—C49	120.79 (19)
C3—C2—H2	126.5	C47—C48—H48	119.6
C1—C2—H2	126.5	C49—C48—H48	119.6
C2—C3—C4	107.00 (19)	C50—C49—C48	118.75 (19)
C2—C3—H3	126.5	C50—C49—H49	120.6
C4—C3—H3	126.5	C48—C49—H49	120.6
N1—C4—C5	125.06 (18)	C49—C50—C51	121.91 (18)
N1—C4—C3	109.77 (18)	C49—C50—O5	118.55 (18)
C5—C4—C3	124.86 (18)	C51—C50—O5	119.51 (18)
C4—C5—C6	125.52 (18)	C50—C51—C52	118.71 (19)
C4—C5—C21	118.37 (17)	C50—C51—H51	120.6
C6—C5—C21	116.05 (17)	C52—C51—H51	120.6
N2—C6—C5	125.73 (18)	C51—C52—C47	121.18 (19)
N2—C6—C7	109.93 (17)	C51—C52—H52	119.4
C5—C6—C7	124.34 (18)	C47—C52—H52	119.4
C8—C7—C6	106.98 (18)	O6—C53—O5	122.91 (19)
C8—C7—H7	126.5	O6—C53—C54	124.4 (2)
C6—C7—H7	126.5	O5—C53—C54	112.71 (18)
C7—C8—C9	107.27 (18)	C55—C54—C59	120.1 (2)
C7—C8—H8	126.4	C55—C54—C53	122.6 (2)
C9—C8—H8	126.4	C59—C54—C53	117.3 (2)
N2—C9—C10	125.67 (17)	C54—C55—C56	119.3 (3)
N2—C9—C8	109.56 (17)	C54—C55—H55	120.4
C10—C9—C8	124.77 (18)	C56—C55—H55	120.4
C11—C10—C9	124.40 (18)	C57—C56—C55	120.0 (3)
C11—C10—C34	119.01 (17)	C57—C56—H56	120.0
C9—C10—C34	116.59 (17)	C55—C56—H56	120.0
N3—C11—C10	124.88 (17)	C58—C57—C56	120.9 (3)
N3—C11—C12	109.46 (17)	C58—C57—H57	119.5
C10—C11—C12	125.61 (18)	C56—C57—H57	119.5
C13—C12—C11	107.13 (18)	C57—C58—C59	119.5 (3)
C13—C12—H12	126.4	C57—C58—H58	120.2
C11—C12—H12	126.4	C59—C58—H58	120.2
C12—C13—C14	107.36 (18)	C58—C59—C54	120.2 (3)
C12—C13—H13	126.3	C58—C59—H59	119.9
C14—C13—H13	126.3	C54—C59—H59	119.9
N3—C14—C15	125.27 (17)	C65—C60—C61	118.7 (2)
N3—C14—C13	109.28 (17)	C65—C60—C20	120.99 (19)
C15—C14—C13	125.44 (18)	C61—C60—C20	120.28 (19)
C14—C15—C16	125.66 (17)	C62—C61—C60	120.9 (2)
C14—C15—C47	117.88 (17)	C62—C61—H61	119.6
C16—C15—C47	116.43 (17)	C60—C61—H61	119.6
N4—C16—C15	125.72 (17)	C63—C62—C61	118.9 (2)
N4—C16—C17	109.40 (17)	C63—C62—H62	120.5
C15—C16—C17	124.87 (18)	C61—C62—H62	120.5
C18—C17—C16	107.19 (18)	C62—C63—C64	121.8 (2)
C18—C17—H17	126.4	C62—C63—O7	118.7 (2)
C16—C17—H17	126.4	C64—C63—O7	119.4 (2)
C17—C18—C19	107.28 (18)	C63—C64—C65	119.1 (2)
C17—C18—H18	126.4	C63—C64—H64	120.5
C19—C18—H18	126.4	C65—C64—H64	120.5
N4—C19—C20	125.76 (18)	C60—C65—C64	120.6 (2)
N4—C19—C18	109.44 (17)	C60—C65—H65	119.7
C20—C19—C18	124.79 (18)	C64—C65—H65	119.7
C1—C20—C19	125.24 (18)	O8—C66—O7	122.9 (2)
C1—C20—C60	117.55 (18)	O8—C66—C67	125.6 (2)
C19—C20—C60	117.20 (18)	O7—C66—C67	111.55 (19)
C22—C21—C26	118.97 (19)	C72—C67—C68	119.7 (2)
C22—C21—C5	120.72 (19)	C72—C67—C66	122.7 (2)
C26—C21—C5	120.25 (19)	C68—C67—C66	117.6 (2)
C21—C22—C23	120.5 (2)	C69—C68—C67	119.9 (3)
C21—C22—H22	119.7	C69—C68—H68	120.1
C23—C22—H22	119.7	C67—C68—H68	120.1
C24—C23—C22	118.9 (2)	C70—C69—C68	120.6 (2)
C24—C23—H23	120.6	C70—C69—H69	119.7
C22—C23—H23	120.6	C68—C69—H69	119.7
C25—C24—C23	121.9 (2)	C69—C70—C71	120.2 (2)
C25—C24—O1	119.1 (2)	C69—C70—H70	119.9
C23—C24—O1	118.9 (2)	C71—C70—H70	119.9
C24—C25—C26	118.8 (2)	C70—C71—C72	119.9 (3)
C24—C25—H25	120.6	C70—C71—H71	120.1
C26—C25—H25	120.6	C72—C71—H71	120.1
C21—C26—C25	120.8 (2)	C67—C72—C71	119.8 (2)
C21—C26—H26	119.6	C67—C72—H72	120.1
C25—C26—H26	119.6	C71—C72—H72	120.1
O2—C27—O1	123.0 (2)	N5—C73—C74	113.2 (3)
O2—C27—C28	125.2 (2)	N5—C73—H73A	108.9
O1—C27—C28	111.80 (18)	C74—C73—H73A	108.9
C29—C28—C33	120.4 (2)	N5—C73—H73B	108.9
C29—C28—C27	123.0 (2)	C74—C73—H73B	108.9
C33—C28—C27	116.6 (2)	H73A—C73—H73B	107.8
C28—C29—C30	118.8 (2)	N6—C74—C73	109.3 (3)
C28—C29—H29	120.6	N6—C74—H74A	109.8
C30—C29—H29	120.6	C73—C74—H74A	109.8
C31—C30—C29	121.0 (2)	N6—C74—H74B	109.8
C31—C30—H30	119.5	C73—C74—H74B	109.8
C29—C30—H30	119.5	H74A—C74—H74B	108.3
C30—C31—C32	120.0 (2)	N6—C75—C76	109.6 (3)
C30—C31—H31	120.0	N6—C75—H75A	109.8
C32—C31—H31	120.0	C76—C75—H75A	109.8
C33—C32—C31	120.0 (2)	N6—C75—H75B	109.8
C33—C32—H32	120.0	C76—C75—H75B	109.8
C31—C32—H32	120.0	H75A—C75—H75B	108.2
C32—C33—C28	119.8 (2)	N5—C76—C75	111.5 (3)
C32—C33—H33	120.1	N5—C76—H76A	109.3
C28—C33—H33	120.1	C75—C76—H76A	109.3
C39—C34—C35	118.02 (18)	N5—C76—H76B	109.3
C39—C34—C10	121.99 (18)	C75—C76—H76B	109.3
C35—C34—C10	119.92 (18)	H76A—C76—H76B	108.0
C36—C35—C34	121.14 (19)		
			
C4—N1—C1—C20	−175.8 (2)	C33—C28—C29—C30	−1.0 (4)
Zn—N1—C1—C20	14.2 (3)	C27—C28—C29—C30	179.8 (2)
C4—N1—C1—C2	0.8 (2)	C28—C29—C30—C31	0.2 (4)
Zn—N1—C1—C2	−169.15 (15)	C29—C30—C31—C32	1.3 (5)
N1—C1—C2—C3	−0.2 (3)	C30—C31—C32—C33	−1.9 (5)
C20—C1—C2—C3	176.5 (2)	C31—C32—C33—C28	1.1 (4)
C1—C2—C3—C4	−0.5 (3)	C29—C28—C33—C32	0.3 (4)
C1—N1—C4—C5	172.7 (2)	C27—C28—C33—C32	179.6 (3)
Zn—N1—C4—C5	−17.2 (3)	C11—C10—C34—C39	−53.7 (3)
C1—N1—C4—C3	−1.1 (2)	C9—C10—C34—C39	125.6 (2)
Zn—N1—C4—C3	168.92 (15)	C11—C10—C34—C35	129.3 (2)
C2—C3—C4—N1	1.1 (3)	C9—C10—C34—C35	−51.3 (3)
C2—C3—C4—C5	−172.8 (2)	C39—C34—C35—C36	−0.9 (3)
N1—C4—C5—C6	1.0 (3)	C10—C34—C35—C36	176.17 (19)
C3—C4—C5—C6	173.9 (2)	C34—C35—C36—C37	0.8 (3)
N1—C4—C5—C21	−175.88 (19)	C35—C36—C37—C38	−0.1 (3)
C3—C4—C5—C21	−2.9 (3)	C35—C36—C37—O3	−176.19 (18)
C9—N2—C6—C5	179.07 (19)	C40—O3—C37—C38	66.6 (3)
Zn—N2—C6—C5	−1.2 (3)	C40—O3—C37—C36	−117.3 (2)
C9—N2—C6—C7	−1.9 (2)	C36—C37—C38—C39	−0.6 (3)
Zn—N2—C6—C7	177.78 (14)	O3—C37—C38—C39	175.36 (17)
C4—C5—C6—N2	9.0 (3)	C37—C38—C39—C34	0.5 (3)
C21—C5—C6—N2	−174.09 (18)	C35—C34—C39—C38	0.3 (3)
C4—C5—C6—C7	−169.9 (2)	C10—C34—C39—C38	−176.78 (18)
C21—C5—C6—C7	7.1 (3)	C37—O3—C40—O4	−3.1 (3)
N2—C6—C7—C8	0.7 (2)	C37—O3—C40—C41	174.80 (18)
C5—C6—C7—C8	179.7 (2)	O4—C40—C41—C42	−173.6 (3)
C6—C7—C8—C9	0.8 (2)	O3—C40—C41—C42	8.6 (4)
C6—N2—C9—C10	−176.66 (19)	O4—C40—C41—C46	5.6 (4)
Zn—N2—C9—C10	3.6 (3)	O3—C40—C41—C46	−172.3 (2)
C6—N2—C9—C8	2.4 (2)	C46—C41—C42—C43	0.0 (6)
Zn—N2—C9—C8	−177.26 (14)	C40—C41—C42—C43	179.2 (4)
C7—C8—C9—N2	−2.1 (2)	C41—C42—C43—C44	0.1 (9)
C7—C8—C9—C10	177.0 (2)	C42—C43—C44—C45	0.8 (9)
N2—C9—C10—C11	−10.6 (3)	C43—C44—C45—C46	−1.9 (8)
C8—C9—C10—C11	170.4 (2)	C44—C45—C46—C41	2.0 (5)
N2—C9—C10—C34	170.12 (18)	C42—C41—C46—C45	−1.1 (5)
C8—C9—C10—C34	−8.9 (3)	C40—C41—C46—C45	179.7 (3)
C14—N3—C11—C10	−174.97 (19)	C14—C15—C47—C48	65.4 (3)
Zn—N3—C11—C10	22.9 (3)	C16—C15—C47—C48	−112.5 (2)
C14—N3—C11—C12	2.7 (2)	C14—C15—C47—C52	−116.5 (2)
Zn—N3—C11—C12	−159.49 (14)	C16—C15—C47—C52	65.5 (2)
C9—C10—C11—N3	−3.6 (3)	C52—C47—C48—C49	0.1 (3)
C34—C10—C11—N3	175.66 (18)	C15—C47—C48—C49	178.19 (19)
C9—C10—C11—C12	179.1 (2)	C47—C48—C49—C50	1.3 (3)
C34—C10—C11—C12	−1.6 (3)	C48—C49—C50—C51	−1.4 (3)
N3—C11—C12—C13	−2.5 (2)	C48—C49—C50—O5	176.48 (19)
C10—C11—C12—C13	175.1 (2)	C53—O5—C50—C49	113.0 (2)
C11—C12—C13—C14	1.3 (2)	C53—O5—C50—C51	−69.0 (3)
C11—N3—C14—C15	177.00 (19)	C49—C50—C51—C52	0.2 (3)
Zn—N3—C14—C15	−20.6 (3)	O5—C50—C51—C52	−177.73 (18)
C11—N3—C14—C13	−1.9 (2)	C50—C51—C52—C47	1.3 (3)
Zn—N3—C14—C13	160.46 (14)	C48—C47—C52—C51	−1.4 (3)
C12—C13—C14—N3	0.4 (2)	C15—C47—C52—C51	−179.52 (19)
C12—C13—C14—C15	−178.5 (2)	C50—O5—C53—O6	−7.2 (3)
N3—C14—C15—C16	4.9 (3)	C50—O5—C53—C54	172.71 (18)
C13—C14—C15—C16	−176.4 (2)	O6—C53—C54—C55	168.5 (3)
N3—C14—C15—C47	−172.86 (18)	O5—C53—C54—C55	−11.5 (3)
C13—C14—C15—C47	5.9 (3)	O6—C53—C54—C59	−8.6 (4)
C19—N4—C16—C15	179.90 (19)	O5—C53—C54—C59	171.5 (2)
Zn—N4—C16—C15	5.9 (3)	C59—C54—C55—C56	1.1 (4)
C19—N4—C16—C17	−1.1 (2)	C53—C54—C55—C56	−175.9 (3)
Zn—N4—C16—C17	−175.12 (14)	C54—C55—C56—C57	−0.1 (5)
C14—C15—C16—N4	3.2 (3)	C55—C56—C57—C58	−1.0 (6)
C47—C15—C16—N4	−179.04 (18)	C56—C57—C58—C59	1.2 (6)
C14—C15—C16—C17	−175.7 (2)	C57—C58—C59—C54	−0.2 (5)
C47—C15—C16—C17	2.1 (3)	C55—C54—C59—C58	−0.9 (4)
N4—C16—C17—C18	1.3 (2)	C53—C54—C59—C58	176.2 (3)
C15—C16—C17—C18	−179.7 (2)	C1—C20—C60—C65	66.7 (3)
C16—C17—C18—C19	−0.9 (3)	C19—C20—C60—C65	−114.4 (2)
C16—N4—C19—C20	−179.2 (2)	C1—C20—C60—C61	−113.3 (2)
Zn—N4—C19—C20	−5.3 (3)	C19—C20—C60—C61	65.7 (3)
C16—N4—C19—C18	0.6 (2)	C65—C60—C61—C62	1.4 (3)
Zn—N4—C19—C18	174.52 (14)	C20—C60—C61—C62	−178.7 (2)
C17—C18—C19—N4	0.2 (3)	C60—C61—C62—C63	−1.1 (3)
C17—C18—C19—C20	180.0 (2)	C61—C62—C63—C64	−0.1 (4)
N1—C1—C20—C19	−1.6 (3)	C61—C62—C63—O7	−176.5 (2)
C2—C1—C20—C19	−177.7 (2)	C66—O7—C63—C62	−103.0 (3)
N1—C1—C20—C60	177.30 (19)	C66—O7—C63—C64	80.5 (3)
C2—C1—C20—C60	1.2 (3)	C62—C63—C64—C65	1.0 (4)
N4—C19—C20—C1	−3.2 (3)	O7—C63—C64—C65	177.4 (2)
C18—C19—C20—C1	177.0 (2)	C61—C60—C65—C64	−0.5 (4)
N4—C19—C20—C60	177.90 (19)	C20—C60—C65—C64	179.6 (2)
C18—C19—C20—C60	−1.9 (3)	C63—C64—C65—C60	−0.7 (4)
C4—C5—C21—C22	89.9 (3)	C63—O7—C66—O8	0.5 (4)
C6—C5—C21—C22	−87.3 (3)	C63—O7—C66—C67	−179.2 (2)
C4—C5—C21—C26	−92.9 (3)	O8—C66—C67—C72	178.3 (3)
C6—C5—C21—C26	89.9 (3)	O7—C66—C67—C72	−2.1 (3)
C26—C21—C22—C23	−2.5 (4)	O8—C66—C67—C68	−1.1 (4)
C5—C21—C22—C23	174.7 (2)	O7—C66—C67—C68	178.5 (2)
C21—C22—C23—C24	0.7 (4)	C72—C67—C68—C69	0.1 (4)
C22—C23—C24—C25	1.5 (4)	C66—C67—C68—C69	179.5 (2)
C22—C23—C24—O1	178.2 (2)	C67—C68—C69—C70	0.7 (4)
C27—O1—C24—C25	−83.3 (3)	C68—C69—C70—C71	−1.2 (4)
C27—O1—C24—C23	99.8 (3)	C69—C70—C71—C72	0.9 (4)
C23—C24—C25—C26	−1.7 (4)	C68—C67—C72—C71	−0.4 (4)
O1—C24—C25—C26	−178.4 (2)	C66—C67—C72—C71	−179.8 (2)
C22—C21—C26—C25	2.3 (4)	C70—C71—C72—C67	−0.1 (4)
C5—C21—C26—C25	−174.9 (2)	C76—N5—C73—C74	−50.0 (3)
C24—C25—C26—C21	−0.2 (4)	Zn—N5—C73—C74	−175.47 (19)
C24—O1—C27—O2	−5.3 (4)	C75—N6—C74—C73	−64.2 (4)
C24—O1—C27—C28	174.4 (2)	N5—C73—C74—N6	57.7 (4)
O2—C27—C28—C29	−162.1 (3)	C74—N6—C75—C76	63.9 (4)
O1—C27—C28—C29	18.2 (3)	C73—N5—C76—C75	48.7 (4)
O2—C27—C28—C33	18.6 (4)	Zn—N5—C76—C75	174.6 (3)
O1—C27—C28—C33	−161.1 (2)	N6—C75—C76—N5	−56.3 (5)

### Hydrogen-bond geometry (Å, º)

*Cg*3 is the centroid of the N3/C11–C14 pyrrole ring. *Cg*10, *Cg*11, *Cg*12, *Cg*15 and *Cg*17 are the centroids of the C21–C26, C28–C33, C34–C39, C54–59 and C67–C72 phenyl rings respectively.

**Table:**

*D*—H···*A*	*D*—H	H···*A*	*D*···*A*	*D*—H···*A*
N5—H5···O4^i^	0.80 (3)	2.15 (3)	2.904 (3)	158 (3)
N6—H6···N1^ii^	0.96 (2)	2.57 (3)	3.434 (4)	151 (3)
C51—H51···O8^iii^	0.95	2.47	3.284 (4)	144
C62—H62···O6^iv^	0.95	2.45	3.339 (4)	155
C39—H39···*Cg*3^v^	0.95	2.81	3.392 (2)	120
C48—H48···*Cg*12^v^	0.95	2.88	3.755 (3)	153
C49—H49···*Cg*17^iv^	0.95	2.90	3.804 (3)	160
C56—H56···*Cg*10^vi^	0.95	2.78	3.623 (3)	147
C64—H64···*Cg*15^iii^	0.95	2.64	3.566 (3)	164
C69—H69···*Cg*11^vii^	0.95	2.95	3.672 (3)	134

Symmetry codes: (i) −*x*+1, −*y*+1, −*z*+1; (ii) *x*+1, *y*, *z*; (iii) −*x*+1, −*y*+2, −*z*+1; (iv) −*x*, −*y*+2, −*z*+1; (v) −*x*, −*y*+1, −*z*+1; (vi) *x*, *y*, *z*−1; (vii) −*x*+1, −*y*+2, −*z*+2.
